# Study of the Effects of Betaine and/or C-Phycocyanin on the Growth of Lung Cancer A549 Cells *In Vitro* and *In Vivo*

**DOI:** 10.1155/2016/8162952

**Published:** 2016-08-22

**Authors:** Rea Bingula, Carmen Dupuis, Chantal Pichon, Jean-Yves Berthon, Marc Filaire, Lucie Pigeon, Edith Filaire

**Affiliations:** ^1^Unité Mixte de Recherche 1019, Centre de Recherche en Nutrition Humaine Auvergne, Université d'Auvergne, 63000 Clermont-Ferrand, France; ^2^CNRS UPR4301, Université Orléans, 45100 Orléans, France; ^3^Greentech SA, Biopôle Clermont-Limagne, 63360 Saint-Beauzire, France; ^4^Centre Jean Perrin, Service de Chirurgie Thoracique, 58 rue Montalembert, 63011 Clermont-Ferrand, France; ^5^CIAMS, Université Paris-Sud, Université Paris-Saclay, 91405 Orsay Cedex, France; ^6^CIAMS, Université d'Orléans, 45067 Orléans, France

## Abstract

We investigated the effects of betaine, C-phycocyanin (C-PC), and their combined use on the growth of A549 lung cancer both* in vitro* and* in vivo.* When cells were coincubated with betaine and C-PC, an up to 60% decrease in viability was observed which is significant compared to betaine (50%) or C-PC treatment alone (no decrease). Combined treatment reduced the stimulation of NF-*κ*B expression by TNF-*α* and increased the amount of the proapoptotic p38 MAPK. Interestingly, combined treatment induced a cell cycle arrest in G_2_/M phase for ~60% of cells.* In vivo* studies were performed in pathogen-free male nude rats injected with A549 cells in their right flank. Their daily food was supplemented with either betaine, C-PC, both, or neither. Compared to the control group, tumour weights and volumes were significantly reduced in either betaine- or C-PC-treated groups and no additional decrease was obtained with the combined treatment. This data indicates that C-PC and betaine alone may efficiently inhibit tumour growth in rats. The synergistic activity of betaine and C-PC on A549 cells growth observed* in vitro *remains to be further confirmed* in vivo. *The reason behind the nature of their interaction is yet to be sought.

## 1. Introduction

Lung cancer causes 28% of global cancer-related deaths and 15% of all diagnosed cancers [1]. It can be subdivided into two broad categories, small-cell lung cancer (SCLC) and non-small-cell lung cancer (NSCLC), the latter being the most common type [2]. The treatment of NSCLC includes surgery, radiotherapy, tyrosine kinase inhibitors, immunotherapy, and platinum-based chemotherapy [3]. With respect to chemotherapy, it appears that natural plant products play an important role, making a considerable contribution to approximately 60% of available chemotherapeutic cancer drugs [4].

The development and progression of cancer involve abnormal changes in DNA methylation which lead to the activation of certain protooncogenes, such as c-Myc, as well as to the inactivation of certain tumour suppressors, such as p16 [5]. It seems that certain dietary components affect the process of carcinogenesis, through DNA methylation. In fact, it has been shown that folate, as methyl group donors, and riboflavin, vitamin B_6_, and vitamin B_12_, as cofactors in one-carbon metabolism, are associated with DNA synthesis and methylation and, hence, may play a role in carcinogenesis and cancer risk; DNA global hypomethylation is associated with lung cancer [6, 7]. Betaine (B) is another major methyl donor [8], which maintains normal DNA methylation patterns [9]. It is naturally found in a variety of food sources including sugar beet, wheat bran, spinach, shrimps, and many others [10]. Humans also obtain betaine from dietary choline, as the liver and kidney are able to oxidize choline into betaine in a two-step enzyme-dependent reaction [10, 11]. Betaine is involved in the synthesis of methionine, which serves as a major supplier of cellular cysteine via a transsulfuration pathway for the synthesis of reduced glutathione that protects the cell from reactive oxygen species [12]. It plays a central role in choline-mediated one-carbon metabolism, cell membrane structural integrity and signalling functions, and neurotransmitter synthesis. Betaine appears to be safe at a daily intake of 9–15 g [12]. Subacute and subchronic rat studies determined that betaine is nontoxic at all doses studied (0–5% of the diet) [13]. Betaine has been inversely associated with the risk of breast cancer [14], nasopharyngeal carcinoma [15], and colorectal cancer [16] and was also shown to decrease lung cancer risk [8]. Moreover, betaine supplementation seems to attenuate oxidative stress [17] and to have an anti-inflammatory effect through NF-*κ*B modulation and antiangiogenic effects [18]. Some articles have shown that betaine is able to inhibit the growth of cancer cells* in vitro* [19, 20] by regulating the expression of protooncogenes and tumour suppressors by stabilizing their methylation patterns [5]. Kim et al. [21] also recently reported that the effects of betaine are linked to the suppression of NF-*κ*B and Akt signal pathways* in vitro* and* in vivo*.

*In vivo*, a decrease in markers of oxidative stress (thiobarbituric acid reactive substances (TBARS), malondialdehyde (MDA)), conjugated dienes, carbonyl proteins, and nitrotyrosine was also noted by Basaran-Küçükgergin et al. [22] following 6 weeks of betaine supplementation (2.5% of chow) in rats. In liver cancer, betaine has a protective effect against the deleterious effects of chemotherapy using cisplatin by limiting the decline of antioxidant enzymes (glutathione disulfide reductase, glutathione peroxidase, catalase, and superoxide dismutase), by eliminating the increase in TBARS concentrations and by attenuating the proinflammatory and apoptotic mediators [23].

Besides betaine, phycocyanin (C-PC) is a protein from the phycobiliprotein family characterized by its intense blue colour and a structure consisting of a protein and nonprotein component known as phycocyanobilin. It is regarded as an ideal food and drug resource, due to its rich protein, lipid, vitamin, mineral, chlorophyll, *β*-carotene, and polysaccharide content. C-PC can be safely added to food, cosmetics, and medicine, with more and more studies confirming that C-PC has many biological activities such as antioxidation, antimutation, antitumour, and antiviral activities, immunity stimulation, hepatoprotective, antiplatelet, and neuroprotective properties and the scavenging of free radicals [24, 25]. Depending on the source, C-PC is classified into three groups: C-PC (from cyanobacteria), R-PC (from red algae), and R-PCII (from* Synechococcus* species) [26].

It appears that C-PC inhibits HeLa cell growth* in vitro*, in a dose-dependent manner by preventing DNA replication [27]. C-PC may induce the apoptosis of colon cancer cell SW480 and laryngeal cancer cell HEP-2 though the mechanism remains unknown [28, 29]. Li et al. [30] have reported that C-PC is an ideal cancer prevention drug as it may inhibit the growth of cancer cells* in vivo* and* in vitro*. Thus, C-PC may become a good substitute for highly toxic conventional chemotherapeutic anticancer drugs in the future [31].

To our knowledge, no studies have investigated the effect of betaine and C-PC on the growth, cell cycle distribution, or apoptosis of A549 cells, human pulmonary epithelial cell line derived from a lung adenocarcinoma, using both an* in vitro* and* in vivo* approach. Therefore, we investigated whether separate or combined treatment with betaine and C-PC would produce an inhibitory effect on the growth of A549 cell line, through a decrease of cell viability and whether this treatment would have an effect on the inflammatory NF-*κ*B pathway. Secondly, we wanted to evaluate whether the combination of C-PC and betaine would influence the p38 MAPK pathway or the cell cycle, as reported in previous studies separately for each compound [18, 32, 33]. Finally, we evaluated the effect of betaine and C-PC on the A549 tumour growth* in vivo* after oral supplementation of nude rats over a 4-week period (following the injection of A549 cells and tumour establishment).

## 2. Materials and Methods

### 2.1. *In Vitro* Study

#### 2.1.1. Treatment Substances

C-PC from* S. platensis* (a gift from Greentech SA, Clermont-Ferrand) in powder form was stored at 4°C protected from light. Both betaine (B2754, Sigma-Aldrich) and C-PC stock solutions were prepared in a phosphate buffer saline (PBS) (Sigma-Aldrich), filtered through a 0.2 *μ*m pore filter and manipulated in a sterile environment. A 10% betaine stock solution (840 mM) was kept at 4°C, while a C-PC stock solution was stored at −20°C and protected from light.

#### 2.1.2. Cell Culture

A549 (ECACC Cell Lines) and A549/NF-*κ*B-Luc (Panomics (ref RC0002)) cell line (A549 cell line with NF-*κ*B inducible promoter for luciferase) of human non-small-cell lung adenocarcinoma were cultured in Dulbecco's Modified Eagle Medium (Sigma-Aldrich), supplemented with 10% Foetal Calf Serum (FCS) (PAA Laboratories), 1% GlutaMAX (Gibco), and 1% Penicillin/Streptomycin (Sigma) at +37°C in 5% CO_2_ atmosphere.

#### 2.1.3. Sample Preparation

The cells (10^4^ cell/well in 96-well plate for cell viability assay, 6·10^4^ cell/well in 24-well plate for all other analysis, 2·10^5^ cell/T25 flask, or 6-well plate for cell cycle analysis and western blot, resp.) were seeded in 100, 400, 5000, and 3000 *μ*L medium, respectively, and cultured overnight. The medium was then removed and different concentrations and combinations of C-PC and betaine solutions were added to the cells and incubated for 24 h and 48 h at +37°C in 5% CO_2_ atmosphere.

#### 2.1.4. Cell Viability Assay

Following betaine and/or C-PC treatment, 3-(4,5-dimethylthiazol-2-yl)-2,5-diphenyltetrazolium bromide (MTT; 20 *μ*L of 5 mg·mL^−1^ solution in PBS) was added to each well containing 100 *μ*L of medium. The cells were then incubated for 4 h at +37°C [34]. MTT converted to formazan was solubilized with acidic isopropanol. The absorbance was measured at 562 nm with a Victor spectrophotometer (Victor 3V Multilabel Plate Reader, Perkin-Elmer).

#### 2.1.5. Western Blot

Cells were washed three times for 5 min with cold PBS; 50 *μ*L of RIPA buffer (Sigma-Aldrich) with phosphatase and protease inhibitor cocktail (Sigma-Aldrich) was added to each well. Plates were incubated for 30 min at +4°C with orbital shaking. The cells were scrubbed, and two well contents were pooled and centrifuged (10 min, 3000 ×g, 4°C). Supernatants were stored at −20°C. On the day of analysis the samples were thawed; approximately 20 *μ*g of each sample (protein quantification kit—BC assays, Interchim, Montluçon Cedex, France) was denatured with Laemmli 5x (10 min, 85°C), loaded on the 12% polyacrylamide gel, and migrated at 130 V (SDS-PAGE) (Mini-PROTEAN® Tetra Cell Systems, Bio-Rad). The transfer to nitrocellulose membrane was done for 80 min under 90 V (Bio-Rad PowerPac HC Power). Following transfer, the membranes were washed once in TBST (tris-buffered saline with Tween 20) and blocked for 1 h in 5% solution of bovine serum albumin (BSA) (Sigma) in TBST. The membranes were washed in TBST three times for 5 min and incubated with agitation overnight at 4°C with primary antibody diluted in 5% BSA solution. After incubation, membranes were washed 3 times for 5 min in TBST and incubated with horseradish peroxidase- (HRP-) coupled secondary antibody in 1% solution of dehydrated milk in TBST for 1 h at room temperature. The membranes were then washed three times for 5 min in TBST and two times in TBS. 3 mL of ECL*™* Prime Western Blotting System (Sigma-Aldrich) was added to the membranes and incubated for 5 min. The blots were visualised by ChemiDoc*™* XRS+ System with Image Lab*™* Software (Bio-Rad). After visualisation of p38 MAPK, the membranes were incubated twice for 10 min with in-house dehybridization buffer, twice for 10 min with PBS, and twice for 5 min with TBST, and, after, the protocol was restarted from the blocking of the membrane for the final detection of the *β*-actin (incubation with the primary antibody was during 6 h on +4°C).

The actin band was detected on the same membrane as the p38 MAPK. The signal quantification was done using Image J software. The primary antibodies used were p38 MAPK rabbit anti-human polyclonal antibody (ADI-KAS-MA009-E, Enzo Life Sciences) 1 : 500 and beta-actin rabbit anti-human polyclonal antibody (PA1-183, Thermo Scientific) 1/1000. The secondary antibody used was goat anti-rabbit IgG (H+L), HRP conjugate (#32460, Thermo Scientific) 1/1000.

#### 2.1.6. RLU (Relative Light Unit) Measurement

A549/NF-*κ*B-Luc cells treated for 24 h with betaine and/or C-PC were washed three times with PBS (Sigma-Aldrich), and DMEM without FBS was added along with human TNF*α* (Miltenyi Biotec) at final concentration of 25 ng·mL^−1^ as an inductor of the NF-*κ*B pathway. Following 9 h incubation, cells were washed once in PBS, trypsinised, mixed with complete medium, and centrifuged for 7 min at 1500 ×g and the dried pellet was stored at −80°C until the day of analysis. The samples were thawed and washed with 500 *μ*L PBS (5 min, 1500 ×g). The pellet was resuspended in 300 *μ*L of Cell Culture Lysis buffer (Promega) with TritonX-100, vortexed for 30 s, and incubated for 10 min at room temperature. Samples were centrifuged for 10 min at 3000 ×g, 60 *μ*L of supernatant was put in the luminometer tubes on ice, and 100 *μ*L mixture of lysis buffer with 10 mM ATP was added to each tube. Luciferase activity was measured using a luminometer (LUMAT LB 9507), normalized to total cell protein using a BCA protein assay kit (protein quantification kit—BC assays, Interchim, Montluçon Cedex, France) and expressed as relative light units (RLU) per mg protein.

#### 2.1.7. Cell Cycle Analysis

A549 were synchronized in G_1_ phase following FBS deprivation followed by thymidine inhibition of DNA synthesis. Cells were first incubated 24 h in DMEM without FBS, followed by 24 h in complete medium with 5 mM thymidine. At that point cells were synchronized in G_1_ phase and were then incubated with C-PC and/or betaine. Following 24 h, cells were collected by trypsinisation, an equal amount of cells from each condition was centrifuged (5 min 3000 ×g), the pellet was gently resuspended in 300 *μ*L of PBS, mixed with 700 *μ*L of precooled 100% ethanol by gently inverting the tube, and stored at −20°C. On the day of analysis, the cells were pelleted (10 min, 5000 ×g), ethanol was removed, and 500 *μ*L of PBS with 20 *μ*L of propidium iodide (50 mg·mL^−1^, Becton Dickinson), 1 *μ*L of RNase (100 mg·mL^−1^), and 0,01% of TritonX-100 were added. The suspension was transferred into FACS tubes (Falcon) and incubated for 30 min at room temperature protected from light. The samples were analysed by flow cytometry (Becton Dickinson FACSort).

### 2.2. * In Vivo* Study: Tumour Rat Model

#### 2.2.1. Cell Culture

A549 cells (ATCC® CCL­185*™*, human lung cancer) were maintained in culture by Cellvax Pharma, in DMEM environment, 10% Foetal Bovine Serum (FBS), 5 mM L-Glutamine, and 1% Penicillin/Streptomycin. A549 cells were grown to a confluence of 80%. For injection, A549 cells were suspended in Matrigel (50 : 50).

#### 2.2.2. Study Design

A total of thirty-four pathogen-free male nude rats (Charles River, France), weighing 239.8 ± 6 g, aged 6-7 weeks, were used for this study. All animal work procedures were approved by the Institutional Animal Care and Use Committee of Maisons-Alfort (France).

Animals were acclimated for one week before initiation of the study. The daily light cycle extended from 7 a.m. to 7 p.m. and room temperature was maintained at 21.6 ± 0.5°C. Throughout the study, rats were allowed to consume food and water ad libitum. Food contained 4% proteins and 3.9% fats, with the remainder being carbohydrates (A03, Society Safe diet, Augy, France). Rats were housed in individual cages.

Following acclimatization, 10^7^ A549 cells were subcutaneously injected into the right flank of the rats.

Ten days following tumour implantation and once mean tumour volumes (MTV) reached 500 mm^3^, all animals were randomly assigned to nonintervention control group (*n* = 8; MTV: 568.8 ± 98.9 mm^3^), betaine treatment group (*n* = 9; MTV: 568.5 ± 133.8 mm^3^), C-PC treatment group (*n* = 8; 597.0 ± 136.4 mm^3^), and betaine + C-PC treatment group (*n* = 8; MTV: 703.4 ± 111.3 mm^3^).

#### 2.2.3. Betaine Treatment

Betaine treatment was administered in drinking water and corresponded to 4% of the daily food intake (2.93 ± 0.3 g by kg of body weight). Betaine supplementation was adjusted every two days, according to the food and water intake of each rat. The daily food, water, and betaine intakes were evaluated for each rat.

#### 2.2.4. C-PC Treatment

C-PC treatment was administered in drinking water and corresponded to 370.0 ± 0.03 mg by kg of body weight. C-PC supplementation was adjusted every two days, according to the food and water intake of each rat. The daily food, water, and C-PC intakes were evaluated for each rat.

#### 2.2.5. C-PC and Betaine Treatment

Betaine and C-PC supplementation was administered in drinking water and corresponded to 4% of the daily food intake for betaine and to 370.0 ± 0.03 mg by kg of body weight for phycocyanin. The daily food, water, Betaine, and C-PC intakes were evaluated for each rat.

#### 2.2.6. Evaluation of Tumour Growth

Tumour growth was measured (tumour length, width, and volume) twice a week by using an external calliper. Tumour volume was calculated by using the following formula: *V* = Length × Width^2^/2. The mean tumour volume (MTV) per group was estimated and given in the graph. The tumour growth data was recorded for each individually identified rat.

The observation period lasted 28 days. All experimental animals were euthanized if during that period the tumour volume reached 4000 mm^3^, as required by institutional guidelines.

At the 29th day, rats were removed from their cages, taken to an adjacent room, and within 30 seconds of removal from the cage were euthanized using anaesthetic (Ketamine: 75 mg·kg^−1^ + Xylazine: 8 mg·kg^−1^) and an aortic puncture. Immediately after euthanization, tumours were excised, weighed, and snap-frozen in liquid nitrogen and stored at −80°C.

## 3. Statistical Analysis

All statistical tests for the* in vivo* experiments were performed using the statistical package programme SPSS 19 (SPSS Inc., Chicago, IL). All values were expressed as the mean ± SE.

Nonparametric analyses (Kruskall-Wallis test and Mann-Whitney *U* test) were used in the statistical computations for tumour weight, tumour growth, food intakes, and body weight.

Statistical significance between groups for* in vitro* studies was assessed by the paired Student's *t*-test (*n* = 3) or repeated measures one-way ANOVA followed by Tukey's posttest (GraphPad Prism, San Diego, California, USA). Differences were considered statistically significant when *p* < 0.05.

## 4. Results

### 4.1. * In Vitro* Study

#### 4.1.1. Viability of the A549 Cells

**Figure 1:**
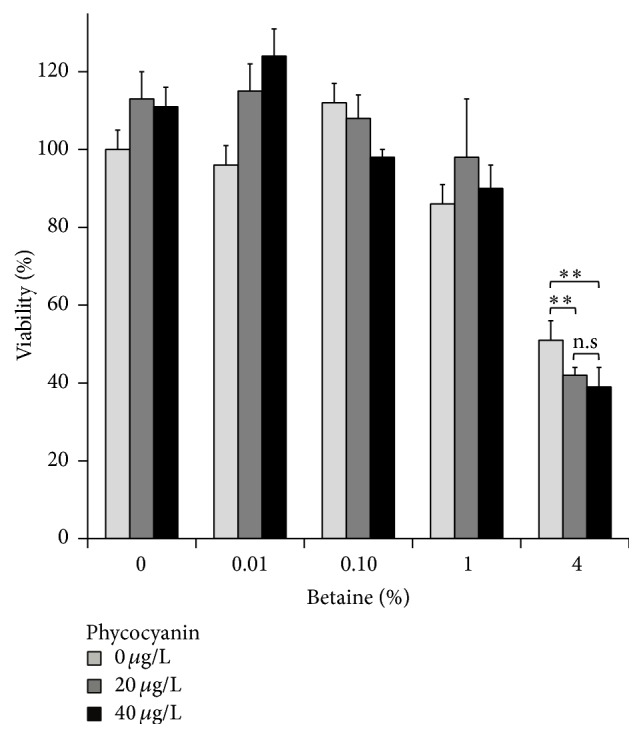
Decrease of cell viability following treatment with betaine and C-PC. Viability of A549 cells following treatment with 0%, 0.01%, 0.1%, 1%, and 4% betaine and 0 *μ*g·L^−1^, 20 *μ*g·L^−1^, or 40 *μ*g·L^−1^ of C-PC. Cell viability was measured using MTT assay after 48 h treatment. Mean ± standard deviation. ^*∗∗*^ *p* < 0.005; n.s stands for not statistically significant. Statistical significance between groups was assessed by the paired Student's *t*-test (*n* = 3).

A549 cells were treated with betaine, C-PC, and a combination of both substances for 48 hours, followed by the viability evaluation through the MTT assay. After 48 h observing the sole betaine treatment, 4% betaine proved to be the most efficient, decreasing the viability of cells by 50%, so this was the one we chose for further application. Treatment with C-PC alone in tested concentrations did not yield a significant viability decrease in any dose or time dependency. On the contrary, combining 4% betaine with different C-PC concentrations decreased the viability by an additional 10–20% (Figure 1). The same effect was also observed for the C-PC concentrations not presented in the figure (data not shown).

#### 4.1.2. Effect of C-PC and Betaine on the Inflammatory NF-*κ*B Pathway

**Figure 2:**
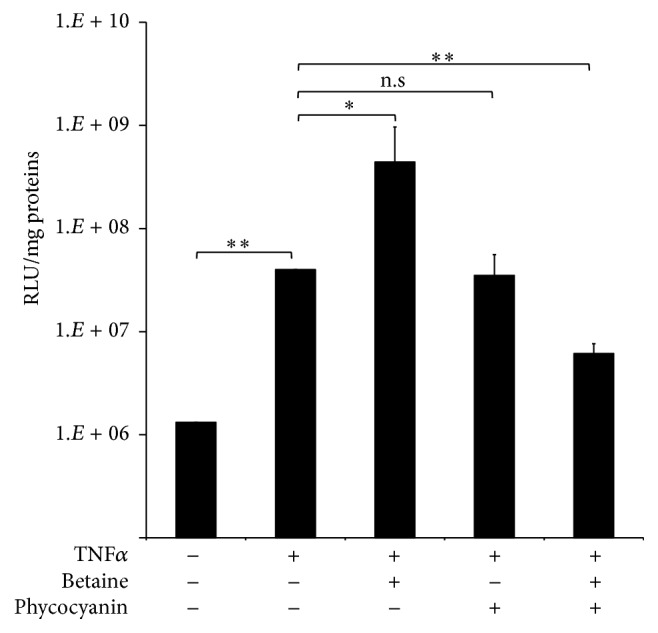
NF-*κ*B expression decrease with combined betaine/C-PC treatment. Measurement of luciferase activity in A549/NF-*κ*B-Luc cells after 24 h treatment with betaine and/or C-PC followed by 9 h treatment with TNF*α*. Mean ± standard deviation. ^*∗*^ *p* < 0.05, ^*∗∗*^ *p* < 0.005; n.s stands for not statistically significant. Statistical significance between groups was assessed by the paired Student's *t*-test (*n* = 3).

To study the effect of betaine and C-PC and their combination on the NF-*κ*B pathway in the context of lung cancer, we used A549/NF-*κ*B-LUC cells (Figure 2). In those cells, luciferase expression is driven by the inducible NF-*κ*B promoter through activation of the NF-*κ*B pathway. As a positive control and as an inducer of the NF-*κ*B promoter activation, A549/NF-*κ*B-LUC cells were treated with 25 ng·mL^−1^ TNF*α* for 9 h.

When cells were first treated for 24 h with C-PC and/or betaine and then further incubated for 9 h with TNF*α*, treatment with 4% betaine alone seemed to elevate the activation of NF-*κ*B. C-PC treatment alone did not have any significant effect while a significant decrease (*p* < 0.05) in activation was only observed when cells were treated with both 20 *μ*g·L^−1^ C-PC and 4% betaine (Figure 2).

#### 4.1.3. Effect of C-PC and Betaine on the p38 MAPK

Western blot was performed to evaluate the expression of p38 MAPK in A549 cells incubated with 4% betaine and/or 20 *μ*g·L^−1^ C-PC. The expression of the p38 MAPK was evaluated by measuring the total p38 MAPK content after 24 h and 48 h and normalization to the *β*-actin. Values of the treatments are expressed as relative densities according to their respective controls.

**Figure 3:**
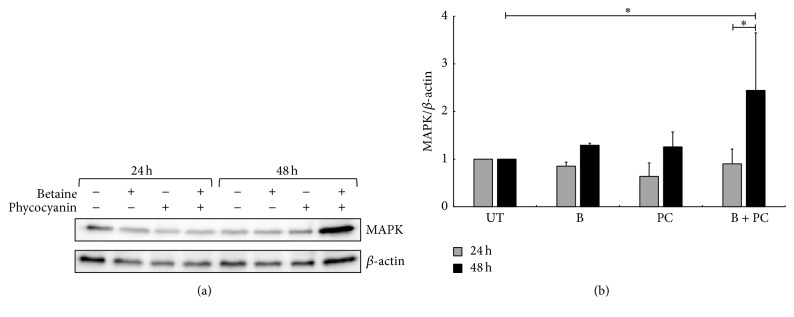
Activation of p38 MAPK pathway on A549 after betaine and/or phycocyanin treatment. Cells were treated with either 4% betaine, 20 *μ*g·L^−1^ of C-PC, or a combination of both molecules for 24 and 48 h. (a) Expression of p38 MAPK in different treatments was monitored by western blot. *β*-actin was used as a loading-control protein. (b) p38 MAPK along with *β*-actin band signals were quantified with the ImageJ software. After normalization with *β*-actin, p38 MAPK values were plotted on the graph as relative values compared to their respective controls. Bars are presented as the mean ± standard deviation. ^*∗*^ *p* < 0.05. Statistical significance between groups was assessed using repeated measures one-way ANOVA followed by Tukey's posttest. UT stands for untreated condition, B stands for betaine, and PC stands for C-PC.

Following 24 h incubation, there was no significant difference in any of the treatment groups (Figure 3(b)—condition B, grey bars). On the other hand, after 48 h, the increase in the expression can be seen in all conditions. While betaine alone and C-PC alone treatments show 1.5-fold increase, which is slight but insignificant, the combination of both gave an approximately 2.5-fold increase in the total p38 MAPK expression (Figure 3(b)—condition B + C-PC black bars). This increase was significant in relation to both 24 h B + C-PC treatment and the 48 h control MAPK expression.

#### 4.1.4. Cell Cycle Analysis

**Figure 4:**
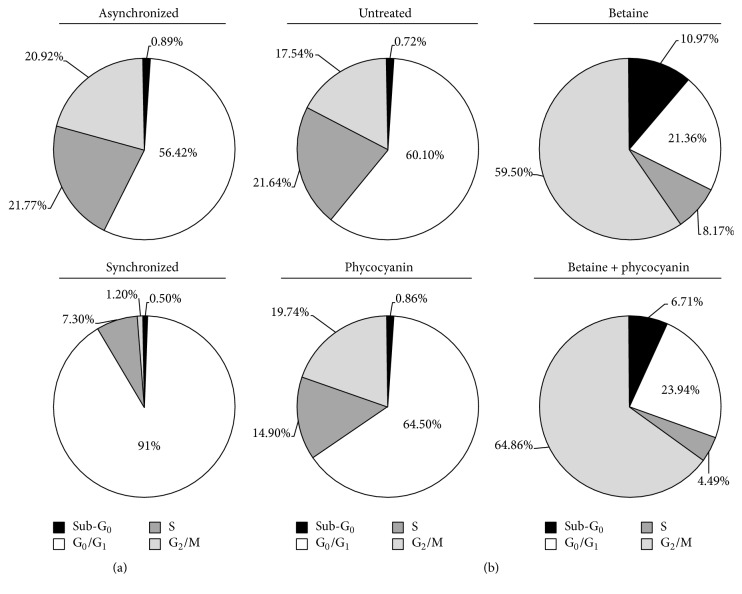
Betaine induced cell cycle arrest in G_2 _phase. Cell cycle study of A549 cells treated with betaine and/or C-PC. (a) First, cells were synchronized in G_1_ phase by serum deprivation followed by thymidine excess (synchronized). (b) Synchronized cells were then treated for 18 h with only medium (untreated), 4% betaine, 80 *μ*g·L^−1^ of C-PC, or a combination of the two treatments (betaine + C-PC). Cell cycles analysed by flow cytometry following propidium iodide staining are shown.

To study the effect of C-PC and betaine and their combination on the cell cycle, A549 cells were synchronized in G_0_/G_1_ cycle by serum deprivation followed by thymidine excess (Figure 4). Cell cycle distribution was evaluated following measurement of DNA content by propidium iodide and monitored by flow cytometry.

Asynchronous A549 showed standard distribution of cells in the different cell cycle phases, with G_0_/G_1_ as the most abundant (56.42%) and approximately equal distribution in S and G_2_/M phase (21.77% and 20.92%, resp.) (Figure 4(a)—asynchronized). The synchronization protocol resulted in 91% of cells in G_0_/G_1_ phase, with almost complete blockage of mitosis (1.20%) and 7% of cells in S phase (Figure 4(a)—synchronized). G_0_/G_1_ synchronized cells were then either untreated or treated with betaine, C-PC, or a combination of both treatments for 18 h (Figure 4(b)).

After 18 h, untreated cells regained a cell cycle distribution similar to asynchronous cells (Figure 4(b)—untreated). 4% betaine treatment (Figure 4(b)—betaine) after 18 h induced an accumulation of cells in G_2_/M phase (59.50% versus 17.54%) and in sub-G_0_ (10.97% versus 0.72%) compared to untreated condition. The treatment with 80 *μ*g·L^−1^ of C-PC lowered the percentage of cells in S phase (14.90% versus 21.64%) in favour of G_1_ phase by 4% (Figure 4(b)—C-PC). The effect of combined treatment was close to that of betaine treatment alone even though C-PC seemed to enhance the accumulation in G_2_/M by 5% as well as the decrease in S phase likely caused by betaine (Figure 4(b)—Betaine + C-PC). Furthermore, while the amount of cells in the sub-Go phase in nontreated cells and in C-PC treated cells remains negligible (<1%), betaine treatment counted 10.97% of sub-G_0_ cells, either apoptotic or necrotic. When betaine was combined with C-PC, this number decreased to 6.71%. In conclusion, it would appear that 4% betaine induced cell cycle arrest in G_2_/M phase and prevented further proliferation of cancer cells, as similarly observed in HeLa cells [35]. 80 *μ*g·L^−1^ of C-PC only slightly influenced the cell cycle by increasing G_1_ [36] and decreasing S phase cells. Interestingly, in combination with betaine, it improved the effect of betaine.

### 4.2. * In Vivo* Study

The body weights of rats were between 372.1 ± 40.2 g and 350.8 ± 42.8 g and were not significantly modified by treatments. Food intake was the same for all groups, suggesting that treatments have no toxic effects.

#### 4.2.1. Effects of Betaine on Tumour Weight and Volume

Table 1 presents tumour volume and weight per group on the day of euthanization. Compared with the control group, tumour weight was significantly lower in betaine, C-PC, and betaine + C-PC treatment group. C-PC treatment induced a higher decrease in tumour weight as compared to betaine treatment. No further higher decrease was noted in the combination treatment group (betaine + C-PC).

**Figure 5:**
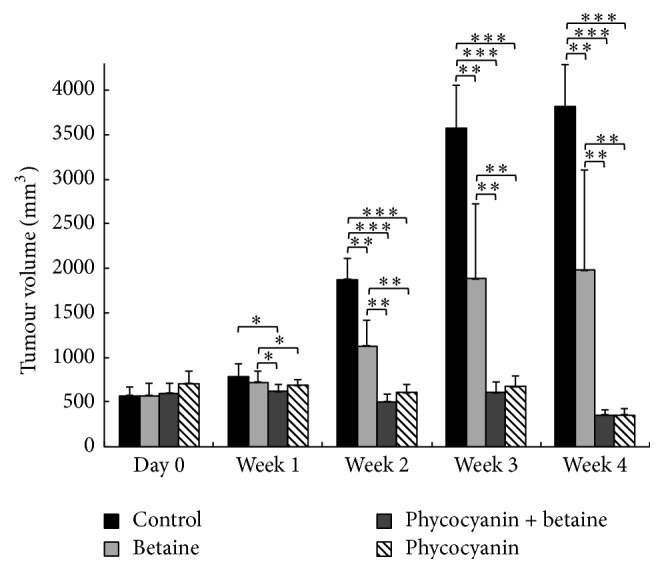
Evolution of mean of tumour volume per week per group, expressed in cubic millimetres (mm^3^). Mean ± standard deviation. ^*∗*^ *p* < 0.05; ^*∗∗*^ *p* < 0.01; ^*∗∗∗*^ *p* < 0.001.

Compared to the control group, a significant difference in tumour volume was noted (Figure 5) after the second week and continued to the end of the experiment.

Following 4 weeks of betaine treatment, tumour volume in the betaine group was 50% lower when compared to the control group, 88.2% lower for the C-PC group, and 86.8% lower for the betaine + C-PC group, respectively.

## 5. Discussion

Cancer, also known as malignant tumour, is a disease that seriously damages health and lives. Recently, scientists have become interested in the potential antitumour effects of nutrients due to their safety and general acceptance. Some data have demonstrated that a large class of natural compounds, including pharmaconutrients, exhibit antitumour activities against selected cancer types [37, 38]. Among them is C-PC, a natural component of edible* S. Platensis *with no toxic side effects that is widely used as an excellent supplement to the human diet [39]. It could inhibit the growth of cancer cells both* in vivo* and* in vitro*. On the other hand, previous studies reported that betaine supplementation has an anti-inflammatory and antiangiogenic effect [18], as well as an anticancer action which may be explained through the regulation of the expression of protooncogenes and tumour suppressors by stabilizing their methylation patterns [40].

We showed that* in vitro* 4% betaine could effectively decrease the viability of A549 lung cancer cells, showing an even better effect if combined with C-PC. The effect observed with betaine correlated to a previous study reporting the decrease in viability following 2% and 4% betaine treatment of Hepa1-6 cells (hepatocellular carcinoma) and HepG2 cells (human hepatoblastoma) [41]. Although there are previous indications that C-PC alone can inhibit cancer cell growth in various concentrations and in a dose-dependent manner by inducing apoptosis [36, 42, 43], we did not observe this. One possible explanation could be the effectiveness of the product, which is highly dependent on the organism of origin, extraction and purification methods, and storage [44, 45].

Apoptosis can be induced through two distinct intracellular pathways mediated either by the death receptor or by the mitochondrial activation, the latter triggered as a response to environmental stress as well as to anticancer drugs [46]. Although weakly activated by growth factors, JNK and p38 MAPK respond strongly to stress signals [47, 48] and chemotherapeutic drugs, and their activation proved necessary for drug-induced apoptosis [49]. p38 MAPK is involved in the mitochondria-mediated apoptotic process and acts one step prior to mitochondrial dysfunction and caspase activation by influencing Bax translocation [50]. Its activation already proved important in the induction of apoptosis in nonsmall lung cancer cells [51]. Our western blot results support the MTT findings of the enhanced effect in combined B + C-PC treatment, showing that there is a greater impact on the expression of p38 MAPK when the two substances are used together, possibly activating apoptosis and reducing cell viability. Conversely, although betaine alone induces a significant viability decrease, there was no significant elevation of MAPK expression, leaving open the possibility of influencing other cellular pathways. Molecular mechanisms of C-PC-induced apoptosis have not been completely elucidated and possible mechanisms such as inhibiting COX-2 activity, reducing the expression of Bcl-2, enhancing the expression of CD59, and activating proteins from the cysteine protease family have been suggested [52]. Recently, Pan et al. [53] reported that C-PC may regulate the expressions of many kinds of proteins in SKOV-3 ovarian carcinoma cells to affect the process of apoptosis, oxidative stress, lipid, and ion transportation and finally inhibit the growth of tumour cells. Although the concentrations used may have been too low to show an effect after administration alone, it is not excluded that this was still enough to add to the effect of betaine by influencing one of the above mentioned pathways.

Disturbance of the cancer cell cycle is one of the therapeutic targets for development of new anticancer drugs [4]. Indeed, cell cycle regulation plays an important role in cell proliferation, differentiation, and apoptosis. In recent years, it has been reported that the dysfunction of cell cycle regulation appears to be closely connected to tumour onset and development. The G_2_/M checkpoint allows the cell to repair DNA damage before entering mitosis and is the most conspicuous target for many anticancer drugs that would cause cell death through the induction of apoptosis [54]. In this study, 4% betaine treatment induced cell cycle arrest in G_2_/M phase, preventing a further proliferation of cancer cells, as similarly observed in HeLa cells [35], in correlation with the observed decrease of cell viability seen in the MTT assay. 11% of the cell distribution found in the sub-G_1_ phase represents the population of cells in apoptosis or necrosis with fragmented nuclear DNA [55]. A successful anticancer compound should kill or incapacitate cancer cells without causing excessive damage to the normal surrounding cells. By inducing cell cycle arrest, the cells are more likely to enter apoptosis, a “clean” death without causing excessive inflammatory cell recruitment into the surrounding tissue through necrotic death, avoiding further damage to the tissue [56]. Incubation of cells with C-PC increased the cells in G_1_ phase, as reported earlier [36, 57]. Wang et al. [58] have also demonstrated that C-PC could inhibit tumour cell proliferation and induce G_2_/M phase arrest in HepG-2 cells. Ying et al. [54] reported that C-PC displayed a significant inhibition effect on the proliferation of human ovarian cancer cell SKOV-3* in vitro*, which was a result of interactions among multiple pathways and signalling molecules. For these authors, interfering with a series of signalling pathways including proteoglycans in cancer, neurotrophin signalling pathway, VEGF signalling pathway, central carbon metabolism in cancer, and p53 pathway and many ending executive factors such as cyclin B1, cyclin-dependent kinase 2 (Cdc2) and Cdc25C, and C-PC influenced the proliferation and apoptosis of cancer cells. A combination with betaine increased the amount of both G_2_/M phase cells and G_1_ phase cells. In this case the sub-G_1_ population was decreased by 4% compared to the betaine treatment alone. Since the sub-G_1_ population contains both necrotic and apoptotic cells, increasing the amount of cells in the cycle arrest that can be guided more easily to apoptosis could bring greater benefit than having a higher amount of the sub-G_1_ phase cells, where necrotic cells are potential triggers for inflammation, especially in an* in vivo* context. The molecular events controlling the G_1_ phase of the cell cycle are determined by a series of phosphorylation events regulated by the expression of specific cyclins, cyclin-dependent kinases (CDK), and CDK inhibitors [59]. The activation of p38 MAPK was reported to lead to the G_2_/M cell cycle arrest by suppressing CDC2 via the phosphatase CDC25 [60], which may be a connection between observed posttreatment increased expression of the p38 MAPK and the cell cycle arrest.

NF-*κ*B, although generally acknowledged as an antiapoptotic and proinflammatory mediator, along with TNF*α* has a complex role in cell signalling which is rather ambiguous [61]. It is already known that, after binding its receptor, TNF*α* induces several responses [62]. Apart from promoting the degradation of I*κ*B and releasing NF-*κ*B transcription factor, it activates two stress-activated MAPK pathways mediated by JNK and p38. Both can have a proapoptotic role [63] by activating caspase-3 of the intrinsic apoptotic pathway, which directly cleaves some of the proteins from the NF-*κ*B pathway, including NF-*κ*B [64]. In contrast to the expected results, luciferase reporting system in A549/NF-*κ*B-Luc cells showed that 4% betaine enhanced TNF*α* activation of NF-*κ*B. However, it is important to keep in mind that this system reports only one side of the TNF*α* signalling pathway (only measuring the promoter activity of the NF-*κ*B gene and not the real content of the protein or mRNA in the cell) and that these same conditions in MTT assay showed a decrease in cell viability. This may lead to the assumption that, in this case, the “death” pathway of TNF*α* is more often activated or that NF-*κ*B here plays a proapoptotic role. In contrast to previous findings [65], C-PC applied alone did not show any inhibitory effect. The reasons may be the different, for example, C-PC purification methods, which influence the protein's properties as already mentioned, or may be due to different concentrations applied. Conversely, when C-PC was combined with 4% betaine it decreased activation of NF-*κ*B promoter (compared to the positive control). The future goal would be to further investigate at which instance and in what manner these two molecules, betaine and C-PC, interfere with the pathways of complex TNF*α* signalling.

To summarize,* in vitro*, betaine alone could inhibit the growth of tumour cells, while the combination of betaine and C-PC showed synergy. However, the effects of C-PC and betaine on lung cancer tumour* in vivo* need further study. In this research we used the NU/NU rat as an animal model to explore the effects of the two substances* in vivo*. Following tumour establishment, its weight and volume were monitored during the supplementation period in each group of rats. Compared with the control group, the tumour weight and volume were significantly reduced in the C-PC and betaine treatment group, respectively (Table 1 and Figure 5), and showed no further decrease in the combination group. In this case we observed no synergistic effect of the combined treatment but rather a strong inhibitory effect of C-PC alone. The beneficial effect of betaine could be due to several reasons. Its antioxidant properties are reported in many diseases and conditions, including oxidative stress induced by ethanol [17], restraint stress [12], and treatment of Parkinson's disease [66]. In mice with colon tumour, betaine specifically inhibited ROS generation and decreased GSSG concentration [21]. The other crucial role of betaine is as a methyl donor, modulating gene expression by targeting different elements of epigenetic mechanisms [67]. Esteller et al. [6] noticed that abnormal promoter DNA methylation was found in all stages of non-small-cell lung cancer, thus bringing betaine and its function into the spotlight. Furthermore, Lee [32] studied mouse hepatocyte H2.35 cells incubated with betaine and showed that betaine treatment leads to an upregulation of mitochondrial respiration and cytochrome c oxidase activity. He suggested that the antiproliferative effects of betaine on cancer cells might be due to enhanced mitochondrial function contributing to a reversal of the Warburg effect. The Akt signalling pathway is known to be associated with promotion of mitochondrial oxidative phosphorylation (OXPHOS) [32] and thus of mitochondrial respiration. Conversely, another study showed that inhibition of heme synthesis and mitochondrial respiration function can arrest the progression of lung cancer cells [68]. Even if the role of mitochondrial respiration is controversial, it seems, in any case, to be one of the key factors in lung cancer progression. Considering the obtained* in vivo* effect of C-PC, we can find the support in earlier publications reporting its beneficial antitumour effects such as the scavenging of free radicals [26] or a concrete growth inhibition in different tumour cell lines, suggesting influence of various mechanisms and cellular pathways [27–30]. One possible explanation for why we did not observe the same inhibitory effect of C-PC* in vitro* could be the fact that the exact metabolism of phycocyanin is yet unknown. Bearing this in mind, it is possible that our* in vitro* concentration of C-PC was simply too low to produce an effect when administered alone.

Observing the obtained results* in vitro*, it is evident that there is some kind of interaction between C-PC and betaine that is still unknown. Since betaine transporters belong to the SLC36 group of proton coupled amino acid symporters that are pH sensitive [69], it is possible that C-PC as a cysteine/methionine rich protein [70] influences the uptake of betaine into the cells by adjusting the pH through the acid-base properties of its amino acid side-chains. Decrease of C-PC fluorescence inside the cells when combined with betaine (seen in the flow cytometry analysis—data not shown) may be explained by C-PC slightly changing the conformation after changing its amino acid charge by donating/accepting H^+^ ions. Also, it might be possible that C-PC interacts with betaine itself, since betaine is an amino acid-like compound, and that changing its charge influences its transport over the cell membrane or interactions inside the cell. However, further investigations are needed to confirm this hypothesis.

## 6. Conclusion

In summary, betaine and C-PC proved to be easily applicable and effective inhibitors of the A549 cell line growth. While in* in vitro* experiments the effect of C-PC was very slight or none, combining it with betaine seemed to enhance an already strong betaine effect. Thus, this combined treatment proved to be an effective inhibitor of the growth of A549 cells by inhibiting the progress of the cell cycle, reducing the cell viability, or enhancing the proapoptotic pathways. However,* in vivo*, both C-PC and betaine alone could successfully inhibit the growth of tumour in rats, and the tumour-suppressing effect was not more evident in the group receiving the combined treatment. Analysis of the methylation pattern following treatments, the oxidative status of cells and the organism, other cellular pathways involved, elucidation of the substance metabolism, and the nature of the interaction between betaine and C-PC, all these things, must still be answered to help us understand the underlying narrative.

## Figures and Tables

**Table 1:** Tumour volume in cubic millimetre (mm^3^) and tumour weight in absolute values (g) and relative values (percentage of the body weight (% BW)) on the day of euthanization per group. Mean ± standard deviation.

	Tumour volume (mm^3^)	Tumour weight (g)	Tumour weight (% BW)
Control	4015 ± 713	2.92 ± 0.47	0.79 ± 0.12
Betaine	1874 ± 1154^*∗∗*^	1.63 ± 0.94^*∗*^	0.48 ± 0.28^*∗∗*^
C-PC	473 ± 162^*∗∗∗*$$$^	0.21 ± 0.06^*∗∗∗*$$$^	0.06 ± 0.02^*∗∗∗*$$$^
C-PC + betaine	528 ± 236^*∗∗∗*$$$^	0.20 ± 0.06^*∗*$$$^	0.06 ± 0.02^*∗∗∗*$$$^

Compared to control group: ^*∗*^ *p* < 0.05; ^*∗∗*^ *p* < 0.01; ^*∗∗∗*^ *p* < 0.001; compared to betaine group: ^$$$^ *p* < 0.001.
